# The effect of age on outcome after intra-arterial treatment in acute ischemic stroke: a MR CLEAN pretrial study

**DOI:** 10.1186/s12883-016-0592-5

**Published:** 2016-05-17

**Authors:** Debbie Beumer, Anouk D. Rozeman, Geert J. Lycklama à Nijeholt, Patrick A. Brouwer, Sjoerd F. M. Jenniskens, Ale Algra, Jelis Boiten, Wouter Schonewille, Robert J. van Oostenbrugge, Diederik W. J. Dippel, Wim H. van Zwam, for the MR CLEAN Pretrial Investigators

**Affiliations:** Department of Neurology and Cardiovascular Research Institute Maastricht (CARIM), Maastricht University Medical Center, Oxfordlaan 10, P.O. Box 5800, 6202 AZ Maastricht, The Netherlands; Department of Neurology, Erasmus MC University Medical Center Rotterdam, Rotterdam, The Netherlands; Department of Neurology, MC Haaglanden, The Hague, The Netherlands; Department of Radiology, MC Haaglanden, The Hague, The Netherlands; Department of Radiology, Erasmus MC University Medical Center, Rotterdam, The Netherlands; Department of Radiology, Nijmegen University Medical Center, Nijmegen, The Netherlands; Department of Neurology and Neurosurgery, Brain Center Rudolf Magnus, Utrecht University Medical Center, Utrecht, The Netherlands; Department of Clinical Epidemiology, Leiden University Medical Center, Leiden, The Netherlands; Department of Neurology, St. Antonius hospital, Nieuwegein, The Netherlands; Department of Radiology, Maastricht University Medical Center, Maastricht, The Netherlands

**Keywords:** Intra-arterial treatment, Older age, Acute ischemic stroke

## Abstract

**Background:**

In recent randomized controlled trials (RCTs) intra-arterial treatment (IAT) has been proven effective and safe for patients with acute ischemic stroke (AIS). So far, there seemed to be no interaction between older age (>80) and main treatment effect. We studied the association of older age with outcome and adverse events after IAT in a cohort of intra arterially treated patients.

**Methods and findings:**

Data from all AIS patients with proven proximal anterior circulation cerebral artery occlusion who were intra arterially treated between 2002 until the start of the MR CLEAN trial were studied retrospectively. Duration of the procedure, recanalization (Thrombolysis In Cerebral Infarction score (TICI)), early neurological recovery (i.e. decrease on NIHSS of ≥ 8 points) after one week or at discharge, good functional outcome at discharge by modified Rankin Scale (mRS ≤ 2) and the occurrence of neurological and non-neurological adverse events were assessed and the association with age was investigated. In total 315 patients met our inclusion criteria. Median age was 63 years (range 22–93) and 17 patients (5.4 %) were over 80. Age was inversely associated with good functional outcome (adjusted Odds Ratio (aOR) 0.80, 95 % CI: 0.66–0.98) for every 10 years increase of age. Age was not associated with longer duration of the procedure, lower recanalization rate or less early neurological recovery. The risk of all adverse events (aOR 1.27; 95 % CI: 1.08–1.50) and non-neurological adverse events (aOR 1.34; 95 % CI: 1.11–1.61) increased, but that of peri-procedural adverse events (aOR 0.79; 95 % CI: 0.66–0.94) decreased with age.

**Conclusion:**

Higher age is inversely associated with good functional outcome after IAT in patients with AIS. However, treatment related adverse events are not related to age. These findings may help decision making when considering treatment of older patients with AIS.

## Background

Stroke is one of the leading causes of death and disability. Risk of stroke increases with age [1]. Higher age is often an exclusion criterion in randomized trials especially in those evaluating acute treatment. This applied to the first trials evaluating the effect of intravenous thrombolysis [2].

Later studies and meta-analyses, however, demonstrated the effect of intravenous alteplase in older patients [3–5]. Recently, the safety and efficacy of intra-arterial treatment (IAT) was established in several large trials [5–9]. Although not all trials included patients aged >80 years [6, 7], in those intervention trials that included older patients treatment effect seemed similar in the young and the old, but the proportions of older patients were small, suggesting selection bias [8, 10, 11]. In older patients, longer procedure time and higher complication rates may be expected, due to technical difficulties such as advanced atherosclerosis and vessel tortuosity. Low quality collateral circulation and brain tissue vulnerability may lead to delayed or diminished neurological recovery.

We studied the association between age and procedure duration, rate of recanalization, occurrence of adverse events, neurological recovery and functional outcome after IAT in patients with acute ischemic stroke (AIS) due to a large vessel occlusion in the anterior circulation. We analysed data from the Multicenter Randomized CLinical trial of Endovascular treatment for Acute ischemic stroke in the Netherlands (MR CLEAN) pretrial registry.

## Methods

### Patients

In this cohort study, we selected patients with a clinical diagnosis of AIS similar to criteria of MR CLEAN [12] from the prospective patient registries of the participating centers. Approval for this retrospective study was obtained by the central medical ethical committee of the Erasmus University Medical Center Rotterdam and because of the retrospective nature of the study no written informed consent was obliged from each participant. Patients were treated intra-arterially in 16 large stroke hospitals in The Netherlands between October 2002 and start of participation of the hospital in the MR CLEAN trial, between 2010 and 2012. We selected all patients with a neurological deficit on the National Institutes of Health Stroke Scale (NIHSS) of at least two points. Intracranial haemorrhage was ruled out by Computed Tomography (CT) or Magnetic Resonance Imaging (MRI). Intracranial arterial occlusion was confirmed by Computed Tomography Angiography (CTA), Magnetic Resonance Angiography (MRA) or Digital Subtraction Angiography (DSA) and was located in the distal part of the internal carotid artery, the middle cerebral artery (M1 and M2 segment), or the anterior cerebral artery (A1 segment). Intra-arterial treatment had to be started within 6 h from onset of symptoms and patients had to be 18 years or older. There was no upper age limit. Patients were excluded if systolic blood pressure was > 185 mmHg or diastolic blood pressure was > 110 mmHg, if blood glucose level was < 2.7 or > 22.2 mmol/L, if intravenous treatment with alteplase was given in a dose exceeding 0.9 mg/kg or 90 mg, or if intravenous alteplase was given despite contraindications such as major surgery, gastro-intestinal bleeding or urinary tract bleeding within the previous 2 weeks, or arterial puncture at a non-compressible site within the previous 7 days. Specific exclusion criteria applied for patients treated mechanically or with intra-arterial thrombolysis. Patients treated with mechanical devices were excluded when there was laboratory evidence of coagulation abnormalities (platelet count < 40*109/l, APTT > 50 s or INR > 3.0). Patients treated with intra-arterial thrombolysis were excluded when the platelet count was < 90 * 109/L, APTT > 50 s, or INR > 1.7.

We obtained all clinical and radiological data of the patients who were prospectively registered in the participating centres and all patient data were anonymized prior to analysis. Missing values of NIHSS were reconstructed with the help of a validated algorithm [13].

### Intervention

Intra-arterial treatment consisted of arterial catheterization with a microcatheter to the level of occlusion. At the level of the occlusion the interventionist could decide to deliver a thrombolytic agent, perform mechanical thrombectomy or both. This choice was left to the discretion of the interventionist. Pre- and post-intervention angiograms were made to assess location and grade of occlusion and final recanalization.

### Outcomes

We tabulated baseline risk factors and results by tertiles of age.

Functional outcome was assessed by means of the modified Rankin Scale (mRS) at discharge. Good functional outcome was defined as a mRS score of 0, 1 or 2. Neurological status after the intervention was evaluated with the NIHSS, assessed at one week after the procedure (or at discharge in case discharge was within the first week). Early neurological recovery was defined as a decrease on the NIHSS of 8 points or more after one week or at discharge compared with baseline NIHSS.

The duration of the procedure was determined by the time interval between the first and last angiographic series plus 5 min. Occlusion status was assessed with the TICI score, at the start and at the end of the procedure. Recanalization was defined as a TICI score of 2b or 3 at the end of the procedure. Digital subtraction angiography images were assessed by one of three experienced neuro-radiologists (PB, GL, SJ). They were blinded for clinical data of the patient, and did not assess angiograms from their own centre.

### Adverse events

We defined symptomatic intracerebral haemorrhage (SICH) as intraparenchymal blood on cranial CT being compatible with neurological deterioration. Symptomatic haemorrhagic transformation was defined as CT detected haemorrhagic transformation of the acute infarct accompanied by neurological deterioration. Recurrent ischemic stroke, stuttering stroke, brain herniation and craniotomy accompanied with neurological deterioration were all clustered as progression of ischemic stroke.

Adverse events were categorized as neurological adverse events, neurological serious adverse events, non-neurological adverse events and peri-procedural adverse events. Neurological adverse events were any symptomatic intracerebral haemorrhage, haemorrhagic transformation, progression of ischemic stroke, intracranial haemorrhage and transient neurological dysfunction including seizures. Neurological serious adverse events were symptomatic intracranial haemorrhage, haemorrhagic transformation of the infarct and progression of ischemic stroke with an increase on the NIHSS of 4 points or more. Non-neurological adverse events, without neurological sequelae, were pneumonia, urinary tract infections, cardiac rhythm disturbances, and other complications. Other complications included pulmonary embolism, cerebral venous thrombosis, bacteraemia, use of antibiotics without a confirmed infection, oral mycosis, delirium, acute kidney failure and falls. Peri-procedural adverse events included development of distal micro-emboli, technical problems including unfolding of stent retriever, iatrogenic damage of arterial vessel wall, groin hematoma and peri-procedural seizures.

### Statistical analysis

We analysed the association of age with good functional outcome (mRS 0–2) with regression models expressed as odds Ratio (OR). Multiple linear regression was used to determine the association of age with the duration of the procedure and the neurological outcome (NIHSS) after the procedure. We analyzed the association of age with recanalization and occurrence of adverse events, with logistic regression models. In all regression models, age was a continuous variable, and effect parameters were expressed per 10 years of age. Precision of effect estimates was described by 95 % confidence intervals. We always adjusted for sex, onset to treatment time, stroke severity at baseline (NIHSS), use of a retrievable stent and presence of intracranial carotid T occlusions. Values missing at random were imputed with their mean or modus. Statistical analyses were carried out with Stata statistical software (release 12.0 College Station, Texas).

## Results

During the study period 529 patients with ischemic stroke were treated with IAT in the participating centers. Of these, 160 had a posterior circulation stroke and were not included in this study. From the remaining 369 patients, we excluded 19 (5.1 %) who underwent IAT more than 6 h after symptom onset, 15 (4.1 %) because of AIS secondary to a surgical procedure, 1 patient (0.3 %) in whom carotid artery dissection was the cause and 1 patient (0.3 %) who had a distal anterior circulation cerebral artery occlusion (M3 segment). We also excluded 2 patients (0.5 %) because of age < 18 years, 4 patients (1.0 %) because systolic blood pressure > 185 mmHg or diastolic blood pressure > 110 mmHg, 11 patients (3.0 %) because of coagulation disturbances and 1 patient (0.3 %) because of pre-treatment glucose level > 22.2 mmol/l.

As a result 315 patients were included. Median age of the included patients was 63 years (range 22–93) and 17 patients (5 %) were older than 80. Median NIHSS was 16 (IQR 12–18) and 245 patients (78 %) were treated with intravenous alteplase prior to IAT (Table 1).

**Table 1:** Baseline characteristics of the included patients

	Overall	Tertile 1	Tertile 2	Tertile 3
	22–93 years	22–55 years	56–70 year	71–93 years
Number of patients	315	106	113	96
Age, median (IQR)	63 (52–72)	48 (40–52)	64 (60–68)	77 (73–80)
Male sex, n (%)	168 (53 %)	52 (49 %)	75 (66 %)	41 (43 %)
Systolic blood pressure, mean (SD)	146 (23)	138 (21)	149 (24)	152 (20)
Diastolic blood pressure, mean (SD)	82 (16)	78 (16)	85 (15)	84 (17)
Stroke severity at admission (NIHSS) mean (SD)^a^	16 (6.0)	15 (5.0)	16 (5.0)	16 (7.0)
Prior treatment with intravenous rtPA, n (%)^b^	245 (78 %)	77 (73 %)	91 (81 %)	75 (80 %)
Time from onset symptoms to intra-arterial treatment (minutes), mean (SD)^c^	93 (58)	96 (70)	87 (67)	77 (53)
Use of stent, n (%)^d^	132 (42 %)	41 (39 %)	52 (46 %)	39 (41 %)
Carotid T top occlusion, n (%)^e^	25 (8.0 %)	11 (10 %)	11 (10 %)	3 (3.0 %)

*SD* standard deviation, *IQR* interquartile range, *ER* emergency room

^a^65 unknown (21 %); ^b^13 unknown (4.1 %); ^c^177 unknown (56 %); ^d^4 unknown (0.6 %); ^e^2 unknown (0.6 %)

IAT consisted of stent thrombectomy in 132 (42 %). The median duration of the procedure was 116 min (IQR 80–153). At the end of the procedure a TICI 2b or 3 was reached in 119 patients (43 %) (see Table 2). SICH occurred in 23 patients (7.0 %) and prevalence of SICH was highest in the third age tertile (10/96, 10 %). Mortality was higher in older patients, 22 % in the third tertile versus 9 % in the first tertile.

**Table 2:** Clinical and radiological outcomes in patients with IAT for acute ischemic stroke caused by proximal arterial intracranial occlusion of the anterior circulation by age

	Overall	Tertile 1	Tertile 2	Tertile 3	P for trend
	22–93 years	22–55 years	56–70 year	71–93 years	
*N* patients	315	106	113	96	
Procedural duration in minutes, median (IQR)	116	125	110	116	0.19
	(80–153)	(81–160)	(78–143)	(82–158)	
Recanalization (TICI 2b and TICI 3)	119 (43 %)	41 (34 %)	45 (38 %)	33 (28 %)	0.46
TICI 0, *n* (%)	38 (14 %)	12 (12 %)	12 (12 %)	14 (17 %)	
TICI 1, *n* (%)	18 (6.0 %)	10 (10 %)	5 (5.0 %)	3 (4.0 %)	
TICI 2a, *n* (%)	103 (37 %)	36 (36 %)	35 (36 %)	32 (39 %)	
TICI 2b, *n* (%)	36 (13 %)	13 (13 %)	17 (18 %)	6 (7.0 %)	
TICI 3, *n* (%)	83 (30 %)	28 (28 %)	28 (29 %)	27 (33 %)	
NIHSS after IAT, mean (SD)	13 (12)	12 (10)	13 (11)	15 (13)	0.14
Neurological recovery (decrease in NIHSS of 8 points or more), *n* (%)	133 (42 %)	47 (44 %)	49 (43 %)	37 (39 %)	
Functional outcome, mRS, *n* (%)					0.03
mRS 0–1, *n* (%)	37 (12 %)	15 (14 %)	9 (8 %)	13 (14 %)	
mRS 0–2, *n* (%)	78 (25 %)	29 (27 %)	27 (24 %)	22 (23 %)	
mRS 0–3, *n* (%)	132 (42 %)	46 (43 %)	49 (43 %)	37 (39 %)	
mRS 0–4, *n* (%)	222 (70 %)	80 (75 %)	82 (73 %)	60 (63 %)	
mRS 0–5, *n* (%)	261 (83 %)	96 (91 %)	92 (81 %)	73 (76 %)	
Death, *n* (%)	51 (16 %)	10 (9 %)	20 (18 %)	21 (22 %)	0.01
All adverse events, *n* (%)	177 (56 %)	46 (43 %)	71 (63 %)	60 (63 %)	0.004
Serious adverse events, *n* (%)	47 (15 %)	13 (12 %)	18 (16 %)	16 (17 %)	0.11
Symptomatic intracranial hemorrhage, *n* (%)	23 (7.0 %)	5 (5.0 %)	8 (7.0 %)	10 (10 %)	
Hemorrhagic transformation infarct, *n* (%)	6 (2.0 %)	2 (2.0 %)	4 (4.0 %)	0 (0.0 %)	
Progression of ischemic stroke, *n* (%)	32 (10 %)	8 (8.0 %)	13 (12 %)	11 (12 %)	
Neurological adverse events, *n* (%)	119 (38 %)	38 (36 %)	48 (42 %)	33 (34 %)	0.60
Asymptomatic intracranial hemorrhage	22 (7 %)	2 (2.0 %)	15 (13 %)	5 (5.0 %)	
hemorrhage, *n* (%)					
Seizures, *n* (%)	16 (5.0 %)	4 (4.0 %)	4 (4.0 %)	8 (8.0 %)	
Non-neurological adverse events, *n* (%)	101 (32 %)	20 (19 %)	40 (35 %)	41 (43 %)	0.002
Pneumonia, *n* (%)	41 (13 %)	9 (8.0 %)	16 (14 %)	16 (17 %)	
Urinary tract infection, *n* (%)	18 (6.0 %)	5 (5.0 %)	5 (4.0 %)	8 (8.0 %)	
Cardiac arrhythmias, *n* (%)	35 (11 %)	3 (3.0 %)	17 (15 %)	15 (16 %)	
Other, *n* (%)	29 (9.0 %)	8 (8.0 %)	9 (8.0 %)	12 (13 %)	

We found a significant inverse association between age and good functional outcome (mRS 0–2) (aOR 0.80; 95 % CI: 0.66–0.98) for every 10 years increase of age. Furthermore, we found a significant association between age and the occurrence of all adverse events (aOR 1.27, 95 % CI: 1.08–1.50) as well as the occurrence of non-neurological adverse events (aOR 1.34, 95 % CI: 1.11–1.61) (Table 3). Age was significantly associated with less peri-procedural adverse events (aOR 0.79, 95 % CI: 0.66–0.94) (Table 4). We found no association between age and neurological recovery after IAT (aOR 0.88, 95 % CI: 0.75–1.04). No associations were observed between age and duration of the procedure (regression coefficient −2.96 95 % CI: −7.35–1.44), recanalization (aOR 0.94, 95 % CI: 0.79–1.11), neurological serious adverse events (aOR 1.22, 95 % CI: 0.96–1.56) and neurological adverse events (aOR 1.05, 95 % CI: 0.89–1.24).

**Table 3:** Age in association with clinical neurological and radiological outcomes. Effect parameters are expressed per 10 years of age

	Association	Unadjusted	Sex adjusted	Fully adjusted
Intervention				
Duration of procedure, minutes	BC	−2.80	−2.80	−2.96
		[−7.10 to 1.45]	[−7.10 to 1.45]	[−7.35 to 1.44]
Recanalization (TICI 2b-3)	OR	0.94	0.94	0.94
		[0.80 to 1.11]	[0.8. to 1.11]	[0.79 to 1.11]
Clinical outcome				
Neurological recovery (decrease on NIHSS > 7 points)	OR	0.92	0.92	0.88
		[0.79 to 1.08]	[0.79 to 1.08]	[0.75 to 1.04]
Functional outcome, mRS 0-2	OR	0.85	0.85	0.80
		[0.71 to 1.01]	[0.71 to 1.01]	[0.66 to 0.98]
Safety outcome				
Peri-procedural adverse events	OR	0.79	0.79	0.79
		[0.67 to 0.93]	[0.67 to 0.93]	[0.66 to 0.94]
All adverse events	OR	1.27	1.27	1.27
		[1.08 to 1.49]	[1.08 to 1.49]	[1.08 to 1.50]
Neurological serious adverse events	OR	1.04	1.04	1.05
		[0.89 to 1.22]	[0.89 to 1.22]	[0.89 to 1.24]
Neurological adverse events	OR	1.04	1.04	1.05
		[0.89 to 1.22]	[0.89 to 1.22]	[0.89 to 1.24]
Non-neurological adverse events	OR	1.37	1.37	1.34
		[1.14 to 1.64]	[1.14 to 1.64]	[1.11 to 1.61]

*BC* beta coefficient, *OR* odds ratio. Fully adjusted means adjusted for; sex, onset to treatment time, stroke severity at baseline (NIHSS), use of a retrievable stent, presence of a carotid T occlusion

**Table 4:** Clinical and radiological outcome in patients younger than 80 years and patients 80 years or over

	<80 years	≥80 years
*N* patients	298	17
Procedural duration in minutes, median (IQR)	121 (55)	108 (54)
Recanalization (TICI 2b and TICI 3)	113 (43 %)	6 (46 %)
Neurological recovery (decrease in NIHSS of 8 points or more), *n* (%)	9 (53 %)	8 (47 %)
Good functional outcome at discharge, mRS 0–2, *n* (%)	75 (25 %)	3 (18 %)
Death, *n* (%)	47 (16 %)	4 (26 %)
All adverse events, *n* (%)	164 (55 %)	13 (76 %)
Non-neurological adverse events, *n* (%)	91 (31 %)*	10 (59 %)*
Neurological adverse events, *n* (%)	113 (38 %)	6 (35 %)
Neurological serious adverse events, *n* (%)	44 (15 %)	3 (18 %)

*p = 0.02

## Discussion

In the present study we show that the likelihood of good functional outcome after IAT was inversely associated with age, whereas we did not find an increased risk for neurological (serious) adverse events after IAT in older patients although the likelihood of developing non-neurological adverse events increased with age. Interestingly, the risk of developing peri-procedural adverse events decreased with increasing age.

Our results are in line with recent studies that also showed lower rates of good clinical outcome in elderly stroke patients treated with IAT. These retrospective non-randomized studies do not allow conclusions about the treatment effect of IAT in older aged patients. Therefore results of randomized controlled trials are needed. Several large randomized controlled trials have investigated the effect of IAT in AIS, but only three of these had no upper age limit [10, 14, 15]. In two of those no effect modification by age was present [8, 10], but a more refined analysis on age has not been performed so far. Selection bias, by including only the relatively healthier older patients in the trials, cannot be excluded.

Our study has several limitations. First, although patients were prospectively registered, all patient data had to be assessed retrospectively from the hospital records. However, we are confident that all intra-arterially treated patients are included and that there are no missing data on important medical information as the occurrence of adverse events, because all centers kept prospective registries of intra-arterially treated patients and our research team had access to all data. Second, we did not systematically register co-morbidity, such as previous myocardial infarction and previous ischemic stroke. However, it is well known that comorbidity increases with age and this may explain some of the excess mortality and poor outcome. Third, the relatively low percentage of recanalization might be explained by the relative low percentage of treatments with latest generation thrombectomy devices, as these devices only entered the market halfway the inclusion period [16]. These so called stent retrievers have a proven better recanalization success rate than the first generation devices [11, 17]. Fourth, the small percentage of patients above 80 years old (5 %) in this cohort suggests a strong selection bias. This might explain the lower peri-procedural adverse event rate in the older patients: exclusion from treatment of patients with hostile vessels is more likely when patients are older. Fifth, the lack of a reference population to compare with and to know if there was a selection bias for the higher aged subjects who underwent IAT. However, the goal of this study was to describe a relation between age and outcome in intra-arterially treated patients to assess whether un upper age limit in randomized controlled trials is indicated. To have the best comparison between age and treatment effect in age in IAT we need the results of randomized controlled trials.

Our study has several strengths: First, we could make use of prospective registries of the participating hospitals in which all IAT treated patients were documented. As such, all patients treated in these centers are included in this study. Second, all stroke intervention centers in the Netherlands participated in this study what makes it very unlikely that more than just a few patients were not registered and included in this study. And thirdly, all participating centers were comprehensive stroke services with a central function for acute stroke treatment for regional hospitals.

## Conclusion

In summary, we confirm that older age is inversely associated with good functional outcome after IAT in patients with AIS. Furthermore age is associated with increased non-neurological adverse events. Our results, however, do not confirm an increased risk of treatment related events nor an increase of neurological adverse events. This might explain the findings in recent RCTs that older age does not interact with treatment effect of IAT. Knowledge about the effect of age on clinical outcome, procedure related features and adverse events of IAT may enhance proper decision making in treatment of older patients with AIS.

### Ethics

Permission fort his study was given by the Medical Ethical Committee of the Erasmus University Medical Center Rotterdam. Due to the retrospective nature of this study no written informed consent was obtained. All patient data were anonymously analyzed.

### Consent to publish

Not applicable.

### Availability of data and materials

All data supporting the findings is contained within the manuscript.

## Abbreviations

AOR: adjusted odds ratio
AIS: acute ischemic stroke
BC: beta coefficient
CT: computed tomography
CTA: computed tomography angiography
DSA: digital subtraction angiography
IAT: intra-arterial treatment
MRI: magnetic resonance imaging
MRA: magnetic resonance angiography
MR CLEAN: multicenter randomized clinical trial of endovascular treatment for acute ischemic stroke in the Netherlands
MRS: modified Rankin scale
NIHSS: National Institutes of Health Stroke Scale
OR: odds ratio
RCT: randomized controlled trial
SICH: symptomatic intracerebral haemorrhage
TICI: thrombolysis in cerebral infarction
